# Impact of Loading Doses of Direct Oral Anticoagulants on Left Ventricular and Left Atrial Thrombus: The LOAD-LVT Study

**DOI:** 10.7759/cureus.114070

**Published:** 2026-08-06

**Authors:** Kyle G Fischer, Zaynab Omisade, Pratham Patel, Taylor Gorski, Evan J Peterson

**Affiliations:** 1 Department of Pharmacy, Ascension Texas, Austin, USA

**Keywords:** direct oral anticoagulants (doacs), left atrial thrombus, left ventricular thrombus (lvt), oral anticoagulation, thrombus formation

## Abstract

Background

Left ventricular (LV) and left atrial (LA) thrombi are associated with significant thromboembolic risk, particularly in the context of myocardial infarction and atrial fibrillation. While direct oral anticoagulants (DOACs) are recognized as alternatives to warfarin, current studies have not evaluated the utility of loading doses, as routinely used in venous thromboembolism (VTE). The LOAD-LVT study aims to determine whether loading doses of apixaban or rivaroxaban influence bleeding risk, thromboembolic events, or thrombus resolution.

Methods and results

A multicenter retrospective cohort study of adult patients with confirmed LV or LA thrombus treated with apixaban or rivaroxaban at five Ascension hospitals in Texas between January 2017 and November 2024 was conducted. Patients were categorized into two groups: receiving a DOAC loading dose (apixaban 10 mg twice daily for seven days or rivaroxaban 15 mg twice daily for 21 days) and receiving standard or renal-adjusted dosing. The primary outcome was the incidence of major and non-major bleeding events. Secondary outcomes included thromboembolic events and thrombus resolution on follow-up imaging at three and six months. A total of 135 patients were included; 23 (17%) received a loading dose. No major bleeding or thromboembolic events were observed in either group. Non-major bleeding occurred in 9% of patients in the loading group versus 4.5% in the standard group (p = 0.404). Of the 135 patients analyzed, 34.8% in the loading dose group and 33.9% in the standard dose group had follow-up imaging. Among those with repeat imaging, complete resolution was documented in 75% of the patients in the loading dose group compared to 86.8% in the standard dose group (p = 0.807). No significant differences were observed in time to thrombus resolution or outcomes by subgroup.

Conclusion

In this retrospective multicenter cohort study, no significant conclusions could be made regarding loading doses of DOACs vs. standard dosing in assessing thrombus resolution or bleeding in patients with LV or LA thrombus. The optimal dosing strategy remains uncertain and should be further evaluated in prospective, randomized trials designed to assess clinical outcomes such as embolic events, bleeding, and thrombus resolution to answer this important and understudied clinical question.

## Introduction

Left ventricular (LV) thrombus is a recognized complication of acute myocardial infarction (AMI), with significant thromboembolic risks if untreated. Despite advancements in interventional cardiology, the incidence of LV thrombus remains substantial. Current literature recommends vitamin K antagonists (VKAs) for three to six months post AMI [1]. The 2013 American College of Cardiology/American Heart Association ST-elevation myocardial infarction (STEMI) guidelines suggest anticoagulation with VKA is reasonable for patients with STEMI and asymptomatic LV mural thrombi [2]. The 2014 American Heart Association/American Stroke Association Stroke Prevention guidelines suggest treatment with VKA therapy is recommended in most patients with ischemic stroke or transient ischemic attack in the setting of AMI complicated by LV mural thrombus formation [3]. The 2017 European Society of Cardiology (ESC) STEMI guidelines state that oral anticoagulation should be considered [4], and the 2022 consensus statement from the American Heart Association recommends direct oral anticoagulants (DOACs) as a reasonable alternative to VKA in patients with LV thrombus [1]. In the US, approximately one million myocardial infarctions occur annually, with an LV thrombus incidence post-anterior STEMI varying from 4% to 39%, likely higher with routine cardiac magnetic resonance imaging [1]. Additionally, patients with dilated cardiomyopathy face an LV thrombus incidence between 2% and 36%. Thrombus morphology reveals embolization risks up to 22%, particularly within the first month following clot formation, and major adverse cardiovascular events up to 37%, highlighting the ongoing clinical challenge in managing LV thrombus [1,5,6]. Despite the relative frequency of LV thrombus, there is a paucity of data evaluating VKA vs. DOAC and specific dosing regimens when using DOACs in this patient population.

In venous thromboembolism (VTE), higher initial doses of DOACs like apixaban (10 mg twice daily for seven days) and rivaroxaban (15 mg twice daily for 21 days) are used during the acute phase of a clot to provide increased levels of anticoagulation when patients are in a hypercoagulable state [7]. These doses, often referred to as “loading doses,” aim to reduce the risk of early thromboembolic events. However, in the context of LV and left atrial (LA) thrombus, there is limited consensus on the necessity and efficacy of using a loading dose approach. This uncertainty stems from the fact that, in many cases, the thrombus may not be in the acute phase, specifically non-ischemic cardiomyopathy-related LV thrombus and LA thrombus, but could represent an incidental finding of an older, stable clot [1].

Evidence supporting the use of loading doses specifically for LV or LA thrombus remains limited. Trials like RED-VELVET, which assessed DOAC effectiveness in LV thrombus, did not include loading doses of apixaban, leading to questions about whether outcomes would differ with a loading dose similar to those used in VTE cases [8]. Similarly, the X-TRA and the CLOT-AF registry, which evaluated DOAC use for LA thrombus, did not incorporate loading doses, raising further questions about whether such a strategy could expedite thrombus resolution and improve patient outcomes [9].

Limited data exist comparing different dosing regimens, leaving the optimal DOAC dosing for LV and LA thrombus unclear. While randomized trials such as RED-VELVT and NO-LVT have used standard atrial fibrillation dosing, studies like X-TRA and CLOT-AF have also focused on standard dosing strategies for LA thrombus without evaluating loading doses [8-10]. Due to the lack of prospective, randomized studies, the optimal anticoagulation regimen for LV or LA thrombus management is uncertain. The LOAD-LVT study aims to fill this gap.

## Materials and methods

We conducted a multicenter retrospective cohort study at five Ascension Seton hospitals in Texas between January 1, 2017, and November 1, 2024, which was approved by the Ascension Institutional Review Board. Patients were identified through electronic health records using Discern Analytics 2.0. Outcomes were assessed via manual chart review performed by KF and ZO. Adult patients (≥18 years old) with a confirmed diagnosis of LV or LA thrombus by transthoracic echocardiogram (TTE) or cardiac magnetic resonance imaging (cMRI) and treated with apixaban or rivaroxaban were included. Patients were excluded if they had undergone LA appendage occlusion prior to thrombus resolution, were pregnant or breastfeeding, or were already receiving DOACs at the time of thrombus diagnosis for other indications.

Patients were divided into two groups based on whether they received a loading dose of a DOAC. A loading dose was defined as apixaban 10 mg twice daily for seven days or rivaroxaban 15 mg twice daily for 21 days. Standard or renal-adjusted dosing without a loading dose was considered standard therapy.

The primary outcome was the incidence of major and clinically relevant non-major bleeding events, as defined by the International Society on Thrombosis and Hemostasis (ISTH). Major bleeding criteria include clinically overt bleeding that was fatal or associated with any of the following: (a) a fall in hemoglobin level of 2 g/dL or more or documented transfusion of at least two units of packed red blood cells; (b) involvement of a critical anatomical site (intracranial, spinal, ocular, pericardial, articular, intramuscular with compartment syndrome, retroperitoneal). Clinically relevant non-major bleeding was defined as clinically overt bleeding that led to hospitalization, medical/surgical intervention, or a change in anticoagulation management, but did not meet the definition of major bleeding, occurring during treatment and up to six months of follow-up. Secondary outcomes included thromboembolic events (stroke or systemic embolism) and thrombus resolution (complete, partial, or persistent) at three and six months. Subgroup analyses were performed based on thrombus location (LV vs. LA), morphology (apical, mural, laminar), size (<10 mm vs. ≥10 mm), and heart failure classification.

Descriptive statistics summarized baseline demographics. Continuous variables were assessed using Student’s t-test or Wilcoxon rank-sum test as appropriate based on normality (Shapiro-Wilk test or skewness/kurtosis test). Categorical variables were analyzed using chi-square or Fisher’s exact test. Time-to-event outcomes were evaluated using Kaplan-Meier analysis with log-rank tests and Cox proportional hazards modeling. Analyses were performed using Stata (StataCorp LLC, College Station, TX). Based on published data, the incidence of major bleeding with standard anticoagulation therapy (i.e., apixaban) is approximately 3%. To detect a clinically significant increase in major bleeding events to 6% in the loading dose group, with a two-sided alpha level of 0.05 and 80% power, it was calculated that 745 patients per group would be needed.

## Results

A total of 445 patients were reviewed, and 135 patients met the inclusion criteria between January 2017 and November 2024, with 23 (17%) receiving a loading dose of apixaban or rivaroxaban and 112 (83%) receiving standard dosing. Out of the 310 excluded, reasons included being discharged on warfarin, no initial imaging available, concurrent VTE, Watchman procedure within the last 30 days, or on a DOAC prior to LA or LV thrombus finding. Baseline demographics and clinical characteristics are shown in Table 1. The mean age of the study population was 61.2 ± 12.7 years, with no significant difference between groups. Most patients were male (loading group: 65.2%, standard group: 75.9%), and the median body weight and BMI were similar between groups.

**Table 1:** Baseline characteristics. BMI: body mass index; kg: kilogram; Scr: serum creatinine; CrCl: creatinine clearance; ECHO: echocardiogram; MRI: magnetic resonance imaging; HFrEF: heart failure with reduced ejection fraction; HFmrEF: heart failure with mildly reduced ejection fraction; HFpEF: heart failure with preserved ejection fraction; NSTEMI: non-ST-segment elevation myocardial infarction; STEMI: ST-segment elevation myocardial infarction.

Variables	Loading dose group (n = 23)	Standard dose group (n = 112)
Age (years), mean (SD)	58 (14)	62 (13)
Male, n (%)	15 (65.2)	85 (75.9)
Weight (kg), median (IQR)	84.6 (72.1-96.8)	85.4 (69.4-98.0)
BMI (kg/m^2^), median (IQR)	29.0 (25.9-34.5)	28 (23.7-32.7)
Active smoking status, n (%)	8 (34.8)	42 (37.8)
Laboratory values		
Scr (mg/dL), median (IQR)	1.3 (1.0-1.5)	1.20 (0.90-1.5)
CrCl (mL/min), median (IQR)	75 (53.1-102.9)	68.9 (54.1-95.0)
Hemoglobin (g/dL), mean (SD)	13.0 (2.48)	13.8 (2.0)
Parenteral anticoagulation use, n (%)		
Enoxaparin	4 (17.4)	22 (19.6)
Heparin	17 (73.9)	55 (49.1)
None	2 (8.7)	35 (31.3)
Imaging, n (%)		
ECHO	22 (95.7)	99 (88.4)
MRI	1 (4.3)	13 (11.6)
Heart failure classification, n (%)		
HFrEF	16 (69.6)	82 (73.9)
HFmrEF	4 (17.4)	11 (9.9)
HFpEF	3 (13.0)	15 (13.5)
Not available	0 (0.0)	3 (2.7)
History of NSTEMI or STEMI, n (%)	3 (13)	20 (17.8)
Hypercoagulable state, n (%)	0 (0)	1 (0.9)
History of stroke, n (%)	2 (8.7)	10 (8.9)

Creatinine clearance (CrCl) at baseline was also comparable (CrCl median (IQR): 75.0 (53.1-102.9) mL/min vs. 68.9 (54.1-95.0) mL/min in loading vs. standard groups). Hemoglobin levels were similar in both the loading and standard dose groups (mean: 13.0 ± 2.48 g/dL vs. 13.8 ± 2.0 g/dL). Of the 23 patients in the loading dose group, 91% received parenteral anticoagulation, with a mean duration of four days. Of the 112 patients who received standard dosing, 69% received parenteral anticoagulation, with a mean duration of four days. There were no statistically significant differences in the use of parenteral anticoagulants or imaging modality between groups. Regarding patients on anticoagulation and antiplatelet therapy, in the loading dose group, 11 were on concomitant antiplatelet therapy, with the breakdown of eight on aspirin, two on ticagrelor, and one on clopidogrel. The standard dosing group had 70 patients on concomitant antiplatelet therapy, with the breakdown of 50 on aspirin, 19 on clopidogrel, and one on ticagrelor.

Thrombus and anticoagulation characteristics are summarized in Table 2. Patients in the standard dosing group who had an LV thrombus, 20 were in the setting of a non-ST-segment elevation myocardial infarction (NSTEMI), and nine were in the setting of a STEMI. The remaining were in the setting of heart failure or incidental finding. In the loading dose group, four were in the setting of an NSTEMI, and the remaining were in the setting of heart failure or incidental finding. The distribution of thrombus type, choice of DOAC (apixaban vs. rivaroxaban), LV thrombus morphology, and thrombus size did not differ significantly between groups. The majority of patients in both groups had LV thrombus (loading: 73.9%, standard: 74.1%). All patients with left atrial appendage (LAA) or LA thrombus had atrial fibrillation. Among patients with reduced ejection fraction, 69.6% in the loading group and 73.9% in the standard group had heart failure with reduced ejection fraction (HFrEF), with no significant differences in the distribution of heart failure with mildly reduced ejection fraction (HFmrEF) and heart failure with preserved ejection fraction (HFpEF) subtypes.

**Table 2:** Thrombus characteristics. LAA: left atrial appendage; LA: left atrium; LV: left ventricle; mm: millimeter.

Variables	Loading dose group (n = 23)	Standard dose group (n = 112)	P-value
Type of thrombus, n (%)			
LAA	5 (21.7)	25 (22.3)	1.0
LA	1 (4.3)	4 (3.6)	
LV	17 (73.9)	83 (74.1)	
Anticoagulation therapy, n (%)			
Apixaban	21 (91.3)	89 (79.5)	0.246
Rivaroxaban	2 (8.7)	23 (20.5)	
LV thrombus type, n (%)			
Apical	14 (60.9)	66 (58.9)	0.720
Mural	1 (4.3)	13 (11.6)	
Laminar	0 (0)	1 (0.9)	
Not available	8 (34.8)	32 (28.6)	
Thrombus size, n (%)			
<10 mm	2 (8.7)	8 (7.1)	0.941
>10 mm	11 (47.8)	53 (47.3)	
Not available	10 (43.5)	51 (45.5)	

For the primary outcome, no major bleeding events were reported in either group (Table 3). Non-major bleeding occurred in two patients (9%) in the loading group and five patients (4.5%) in the standard group (p = 0.404). No thromboembolic events, including stroke or systemic embolism, occurred in either group during the follow-up period.

**Table 3:** Primary and secondary outcomes.

Outcome	Loading dose group (n = 23)	Standard dose group (n = 112)	P-value
Incidence of bleeding events, n (%)			
Major bleeding event	0	0	0
Non-major bleeding event	2 (9)	5 (4.5)	0.404
Incidence of thromboembolic events, n (%)			
Stroke	0	0	-
Systemic embolism	0	0	-
Thrombus resolution, n (%)			
Complete	9 (39.1)	34 (30.3)	0.807
Partial	2 (8.7)	4 (3.6)	0.432
Persistent thrombus	2 (8.7)	3 (2.7)	0.531
No follow-up imaging	10 (43.5)	71 (63.4)	1.00
Time to thrombus resolution, n (%)			
Within three months	5 (21.7)	17 (15.2)	1
Within six months	4 (17)	12 (10.7)	0.736

Follow-up imaging was available in approximately 35% of patients in each group to assess for thrombus resolution. Outcomes were similar between groups. Among patients with available follow-up imaging, complete resolution occurred in 69% in the loading group and 82.9% in the standard group (p = 0.807). Rates for partial thrombus resolution were 15.4% vs. 9.6%, and rates for persistent thrombus were 15.4% vs. 7.3%, all non-significant. The majority of patients had no follow-up imaging documented (43.5% in the loading group vs. 63.4% in the standard group, p = 1.0). Time to thrombus resolution within three months occurred in five patients (38.5%) in the loading group and in 17 patients (45.5%) in the control group, both non-significant; there were no significant differences in resolution rates at six months either.

## Discussion

The use of loading doses is standard practice in the management of acute VTE to achieve therapeutic anticoagulation rapidly during the initial high-risk period. However, the clinical benefit of loading doses for LV or LA thrombus remains unestablished. This retrospective multicenter cohort study is, to our knowledge, the first to evaluate the impact of loading versus non-loading doses of DOAC’s for the treatment of LV or LA thrombus. Among the key findings was that there were no statistically significant differences between the loading-dose and standard-dose groups across all outcomes evaluated. This could be attributed to the low number of included patients in this study, which led to insufficient power, and the lack of follow-up imaging readily available in this retrospective study.

Prior studies, including the RED-VELVET, X-TRA, NO-LVT, and CLOT-AF registries, used standard DOAC dosing without loading strategies [8-10]. In RED-VELVET, the DOAC treatment was associated with a higher risk of stroke or systemic embolism vs. warfarin (HR, 2.71; 95% CI, 1.31-5.57; p = 0.01). This study did not track bleeding events as a primary outcome but reported eight bleeding events in the DOAC arm vs. 19 in the warfarin arm [8]. The NO-LVT study found that complete LV thrombus resolution occurred more frequently in the rivaroxaban group (71.79%, 76.92%, and 87.17% at one, three, and six months) compared to the warfarin group (47.5%, 67.5%, and 80%; p = 0.03, 0.35, and 0.39). Major bleeding events occurred in 5.1% of the rivaroxaban group vs. 15% of the warfarin group (p = 0.11) [10]. Jones and colleagues published a single-center cohort study comparing the efficacy and safety of DOACs vs. warfarin in LV thrombus post AMI. LV thrombus resolution rates were 70.7% in the DOAC group vs. 48.3% in the warfarin group at first follow-up imaging (p = 0.04). Major bleeding events occurred in 0% of the DOAC group vs. 6.7% in the warfarin group (p = 0.03) [11]. Based on the analysis of the X-TRA and CLOT-AF studies published by Lip et al. in 2016, rivaroxaban appeared to be a potential option for LA/LAA thrombus resolution in patients, with outcomes comparable to warfarin [12]. Furthermore, a recent meta-analysis by Koeckerling and colleagues compared the efficacy and safety of warfarin and DOACs in the treatment of LV thrombus and found no significant differences between warfarin and DOACs in thrombus resolution, all-cause mortality, stroke, and major and nonmajor bleeding [13].

Moreover, our findings regarding thromboembolic events could not be compared with those of prior studies, as none were observed with either regimen in our study. Thrombus resolution by 90 or 180 days was similar between groups, suggesting no added benefit of a loading approach in accelerating thrombus clearance. No major bleeding or embolic events occurred in either group. Non-major bleeding was more frequent in the loading group (9% vs. 4.5%), though this difference was not statistically significant and was not powered to detect a difference.

Current guidelines for LV thrombus recommend three to six months of anticoagulation without specifying a specific agent or dosing regimen. In this retrospective study, use of a loading dose did not reduce thrombotic events, so omission may be reasonable for most patients. Whether a loading dose improves outcomes in selected cases such as early, large, mobile thrombi or recent myocardial infarction remains unknown.

Strengths and limitations

This study included patients from multiple hospitals within the same health system, improving generalizability of findings. It is also the first to specifically examine the use of loading doses in LV or LA thrombus treatment. However, several limitations must be acknowledged. The retrospective design limits conclusions regarding causality and introduces potential for confounding. The number of patients in the loading-dose group is relatively low compared with the standard-dose group. This variation in sample size may have also contributed to the insignificant results. Unmeasured patient characteristics or clinical scenarios may have led providers to select a particular dosing regimen. The majority of patients in the study had LV thrombus; patients with LA thrombus were underpowered for evaluation. Over 65% of patients had no follow-up imaging readily accessible in the electronic medical record (EMR), limiting accurate assessment of thrombus resolution. The lack of access to outpatient networks and outpatient EMR data could have led to missed follow-up outcomes. Thrombotic or bleeding events that occurred after discharge were not captured if the patients were treated at a different hospital system. Additional limitations include incomplete documentation of cardiac history and variability in imaging interpretation across sites. The sample size was underpowered to detect differences in uncommon outcomes such as systemic embolism or major bleeding. Lastly, medication adherence after discharge could not be measured.

## Conclusions

In this retrospective multicenter cohort study, no definite conclusions could be drawn regarding the comparative effectiveness or safety of loading-dose versus standard-dose DOACs for LV or LA thrombus, as the study was underpowered. The unequal sample sizes between the treatment groups may have further limited the ability to detect statistically significant differences in thrombus resolution or bleeding outcomes. Consequently, the optimal DOAC dosing strategy for this patient population remains uncertain. Prospective, adequately powered randomized controlled trials are needed to evaluate clinically meaningful outcomes, including thrombus resolution, embolic events, and bleeding, to address this important and understudied clinical question.
